# A Rare Case of Pleural Deciduoid Mesothelioma With Histological, Immunohistochemical, Fluorescent In Situ Hybridization, and Ultrastructural Findings

**DOI:** 10.7759/cureus.113332

**Published:** 2026-07-24

**Authors:** Hiroshi Sonobe, Rika Omote, Eito Niman, Kenji Takahashi, Kazuki Nabeshima

**Affiliations:** 1 Department of Diagnostic Pathology, National Hospital Organization (NHO) Fukuyama Medical Center, Fukuyama, JPN; 2 Department of Thoracic Surgery, National Hospital Organization (NHO) Fukuyama Medical Center, Fukuyama, JPN; 3 Department of Diagnostic Pathology, Pathological Diagnosis Center, Fukuoka Tokushukai Hospital, Fukuoka, JPN

**Keywords:** deciduoid mesothelioma, fluorescent in situ hybridization, immunohistochemistry, pleura, ultrastructure

## Abstract

Owing to the rarity of deciduoid mesotheliomas, few studies have reported detailed morphological analyses. Here, we report a case of deciduoid mesothelioma along with cytological, histological, immunohistochemical, fluorescent in situ hybridization (FISH), and electron microscopic findings.

The patient was a 75-year-old man with a history of asbestos exposure. Computed tomography revealed multiple nodular shadows in the left pleura, and pleural effusion. Pleural effusion cytology revealed large epithelioid tumor cells with high nuclear atypia, suggesting epithelioid mesothelioma. A biopsy revealed that the tumor tissue consisted of sheets of highly atypical, large epithelioid cells of various sizes. Immunostaining revealed that the tumor cells were positive for mesothelial cell markers, including calretinin, Wilms tumor 1, and podoplanin, and showed loss of expression of BRCA1-associated protein 1, methylthioadenosine phosphorylase, and merlin/neurofibromatosis 2. Furthermore, cyclin-dependent kinase inhibitor 2A-FISH revealed a high frequency of homozygous deletions. Based on these findings, the tumor was diagnosed as a deciduoid mesothelioma. Reflecting the histological features, electron microscopy revealed that the tumor exhibited sheets of large epithelioid cells containing round nuclei with single or multiple distinct nucleoli, and intracytoplasmic intermediate filaments; however, microvillous structures were not evident. After the diagnosis was confirmed, the patient was treated with a combination of immune checkpoint inhibitors and chemotherapy. Long-term follow-up is required, although no disease progression was observed seven months after the initiation of therapy.

## Introduction

Mesotheliomas arising from the serous membranes are most commonly found in the pleura (accounting for more than 80% of cases), followed by the peritoneum (approximately 15-20%), with extremely rare occurrence in the pericardium and tunica vaginalis testis (below 1%) [1]. Asbestos is known to be associated with several diseases, including lung cancer, mesothelioma, and plaque formation. The majority of patients with mesotheliomas (approximately 80%) have a history of asbestos exposure; in which asbestos fibers cause abnormal changes in cyclin-dependent kinase inhibitor 2A (CDKN2A) [2]; however, mesotheliomas may actually occur in patients with an unknown history of asbestos exposure [3]. According to the World Health Organization (WHO) classification (2021) [1], mesotheliomas are classified as epithelioid (approximately 60-80%), sarcomatoid (approximately 10-20%), or biphasic mesotheliomas (approximately 10-20%). Biphasic mesotheliomas are defined as comprising at least 10% each of epithelioid and sarcomatoid components. Epithelioid mesotheliomas show diverse histo-architectural and cytomorphological characteristics. The histo-architectural characteristics of tumor cells include adenomatoid tumor-like, solid, and micropapillary patterns, and their cytomorphological distinctions include rhabdoid, small cell, clear cell, signet ring cell, deciduoid, and lymphohistiocytoid mesotheliomas. In addition, mesothelioma with extensive myxoid stroma exists. Among them, epithelioid mesothelioma with deciduoid features (deciduoid mesothelioma) is exceedingly rare and exhibits a morphological pattern similar to decidua-like features of the uterine stroma occurring in a progesterone-dominant state, [1,4,5]. Therefore, deciduoid mesothelioma may be mistaken for ectopic decidua. This tumor was first reported approximately 40 years ago by Talerman et al. [5] In their report, a peritoneal lesion in a 13-year-old girl was initially misdiagnosed as ectopic decidua, and this case was published together with a review of similar cases. Likely owing to the rarity of this malignancy, there remains no consensus regarding its clinical or prognostic evaluation. In addition, few detailed studies have been conducted on the morphological characteristics of this rare deciduoid mesotheliomas. In this paper, we report the results of cytological, histological, immunohistochemical, and CDKN2A-fluorescent in situ hybridization (FISH), and ultrastructural studies in a case of deciduoid mesothelioma arising in the pleura of an elderly man.

## Case presentation

**Figure 1:**
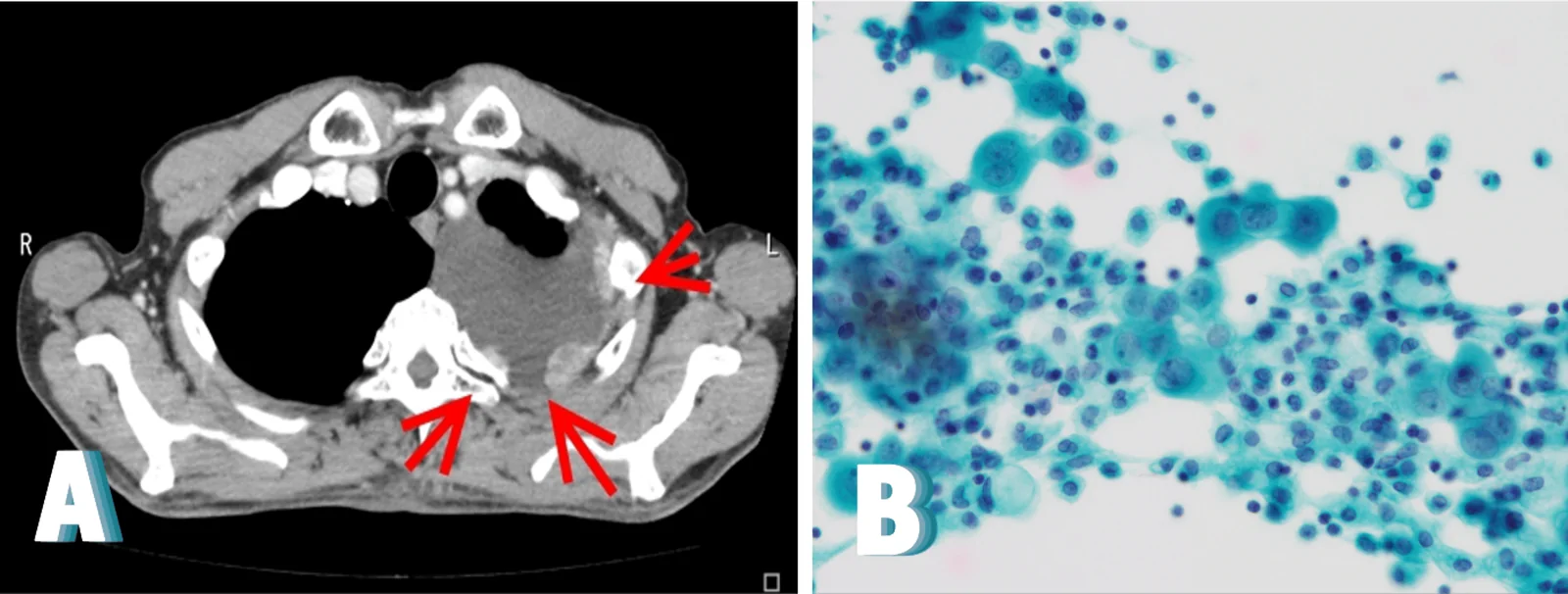
Computed tomography and cytological findings A: Computed tomography shows left pleural multiple nodules and effusion. B: Pleural effusion cytology reveals scattered or small aggregates of large epithelioid tumor cells in a background of numerous inflammatory cells (Papanicolaou stain). (B) Original magnification x 200.

In the present case, the patient was a 75-year-old man with a history of hypertension, emphysema, and chronic heart failure. The patient worked in the painting industry with a history of asbestos exposure. He had a history of smoking 20 cigarettes per day for 45 years (20 to 65 years of age). He was referred to our hospital after chest radiography and computed tomography (CT) scan performed by his family doctor, revealing left pleural effusion and multiple pleural nodules (Figure 1A). Hyaluronic acid in the pleural fluid obtained by thoracentesis was elevated at ≥20×10^4^ng/mL, exceeding the upper limit of detection, and pleural fluid cytology suggested epithelioid mesothelioma. Thoracoscopy and pleural biopsy were performed to confirm the diagnosis.

Pleural effusion and plaques were observed in the pleural cavity. One of the pleural nodules was resected. The pathological diagnosis confirmed mesothelioma, and combination therapy with nivolumab and ipilimumab was initiated. After two courses, enteritis and hepatitis developed due to immune-related adverse events (irAEs), and the therapy was discontinued. Treatment is ongoing for irAEs. Combination therapy has not been resumed, and the patient's condition has remained stable.

**Figure 2:**
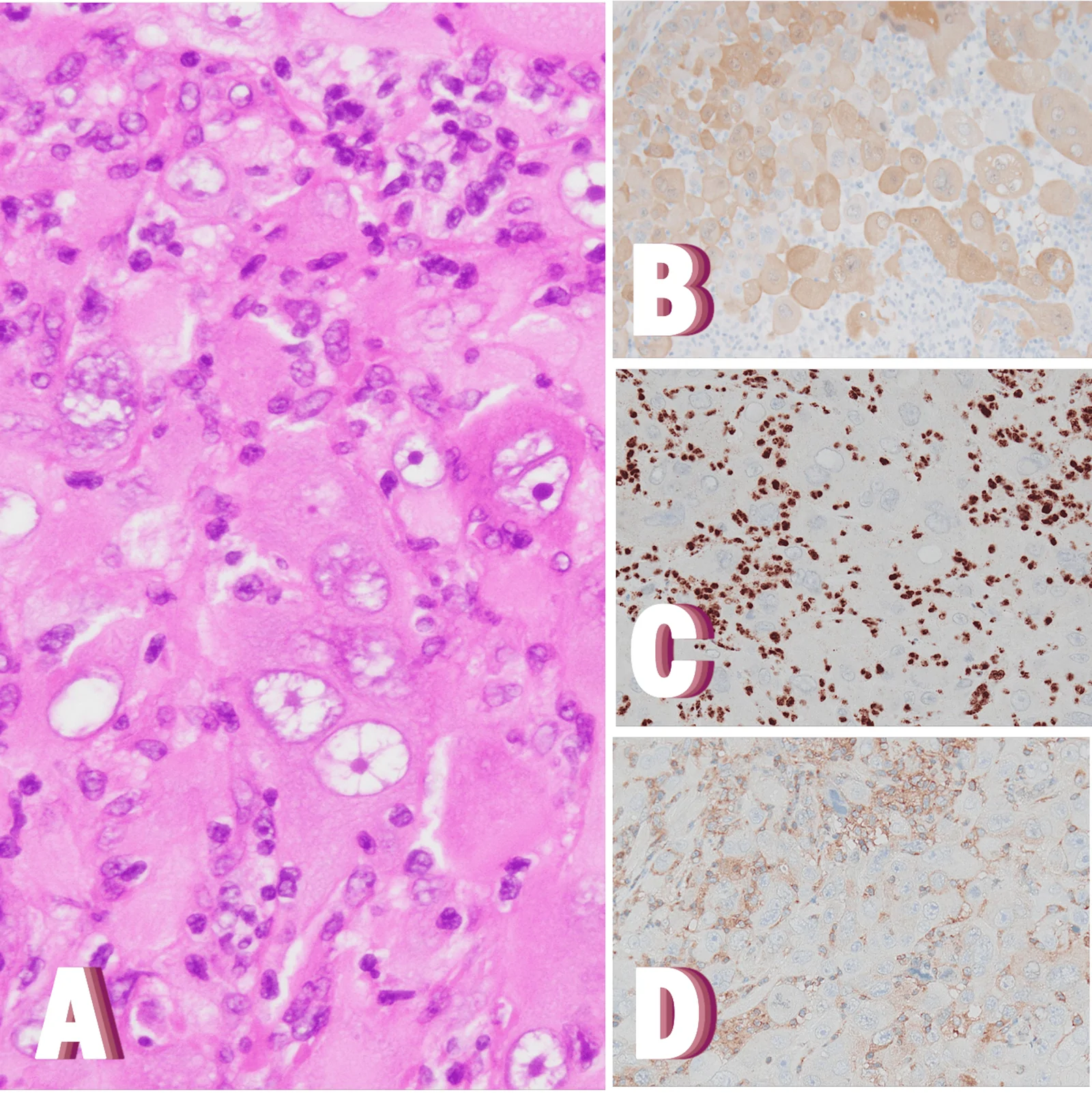
Histological and immunohistochemical findings A: Tumor cells showing abundant homogeneous cytoplasm with round, vesicular, atypical nuclei containing distinct nucleoli, and proliferating in sheets (H&E). B: Tumor cells are positive for calretinin. C: Tumor cells show loss of BAP1. D: Tumor cells show loss of Merlin/NF2. (A) Original magnification x 400, (B-D) Original magnification x 100. H&E: hematoxylin and eosin staining: BAP1: BRCA1-associated protein 1; Merlin/NF2: Merlin/neurofibromatosis 2

Pathologically, effusion cytology showed scattered or small aggregates of large epithelioid tumor cells with abundant cytoplasm in a background of lymphocytes, macrophages, and reactive mesothelial cells. Most nuclei of the tumor cells were round to oval shaped with fine granular chromatin and distinct nucleoli (Figure 1B). Histologically, the tumor cells of various shapes and sizes showed an abundant homogeneous cytoplasm and round, vesicular, highly atypical or pleomorphic nuclei with one or more distinct nucleoli, and proliferated in sheets accompanied by multinucleated tumor cells. In some areas, inflammatory cells, primarily lymphocytes, showed infiltration to varying degrees (Figure 2A). Immunohistochemically, the tumor cells were positive for calretinin (Figure 2B), podoplanin (D2-40), Wilms tumor 1 (WT-1), cytokeratin cocktail AE1/AE3 (AE1/AE3), and vimentin, but not for epithelial membrane antigen (CEA) and thyroid transcription factor 1 (TTF1). Additionally, the tumor cells showed nuclear loss of expression of BRCA1-associated protein 1 (BAP1) (Figure 2C), methylthioadenosine phosphorylase (MTAP), and Merlin/neurofibromatosis 2 (Merlin/NF2) (Figure 2D) (Table 1). These histological and immunohistochemical findings were consistent with deciduoid mesothelioma.

**Table 1:** Immunohistochemical results WT-1: Wilms tumor 1; D2-40: podoplanin; AE1/AE3: cytokeratin cocktail AE1/AE3; CEA: carcinoembryonic antigen; TTF1: thyroid transcription factor 1; BAP1:  BRCA1-associated protein 1; MTAP: methylthioadenosine phosphorylase; Merlin/NF2: Merlin/neurofibromatosis 2.

Markers	Reaction
Calretinin	Positive
WT-1	Positive
D2-40	Positive
Vimentin	Positive
AE1/AE3	Positive
CEA	Negative
TTF1	Negative
BAP1	Loss
MTAP	Loss
Merlin/NF2	Loss

**Figure 3:**
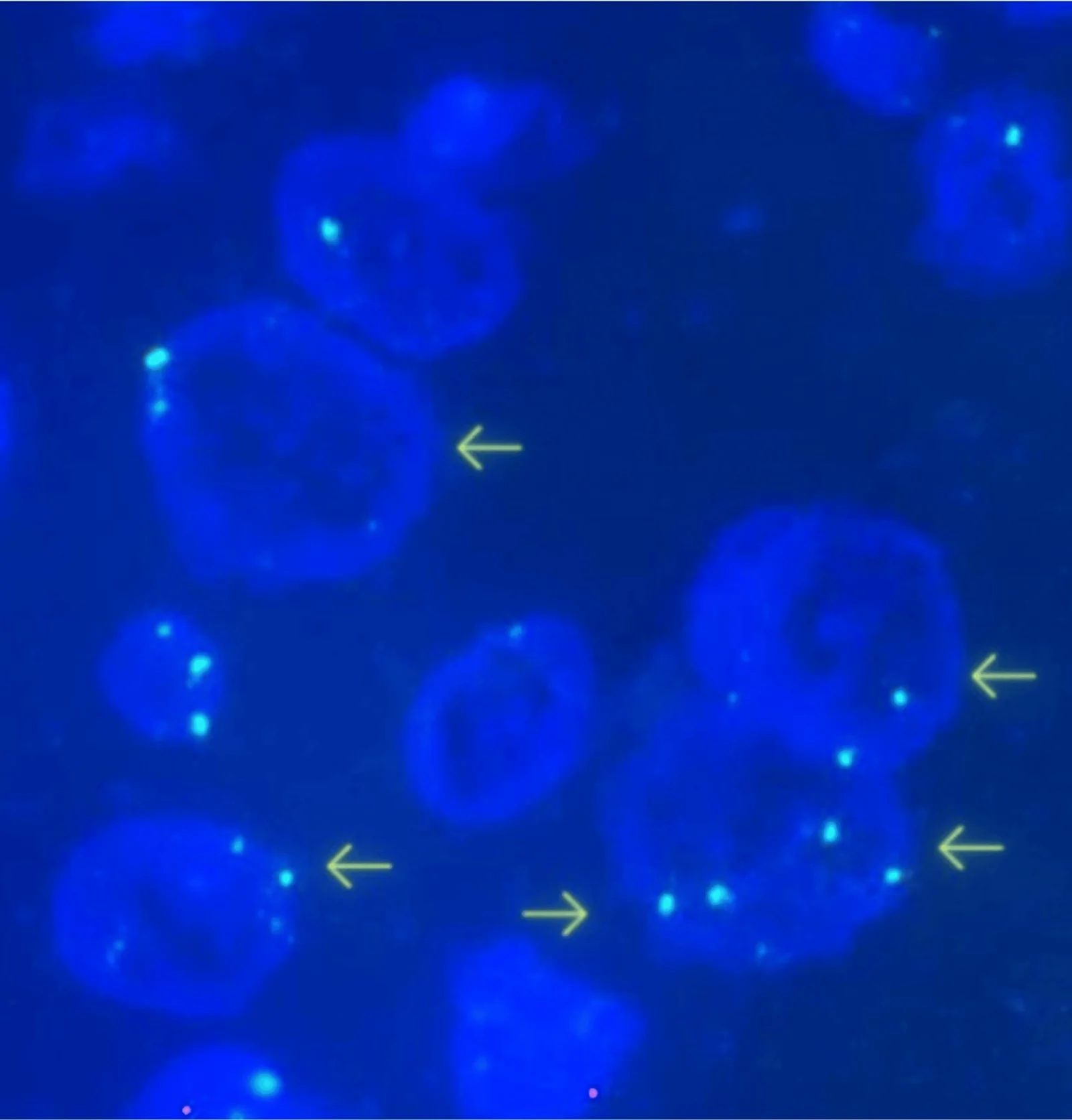
Cyclin-dependent kinase inhibitor 2A-fluorescent in situ hybridization Fluorescent in situ hybridization revealing homozygous deletions of cyclin-dependent kinase inhibitor 2A (yellow arrow) in tumor cells.

FISH for the detection of 9p21, including CDKN2A, revealed not only a high frequency of homozygous deletions (65.8%: 75/114) but also heterozygous deletion (0%: 0/114), one signal of centromere signal (31.6%: 36/114), one pair signal of centromere and 9p21 including CDKN2A (2.6%: 3/114), and a normal pattern (0/114: 0.18%) (Figure 3).

**Figure 4:**
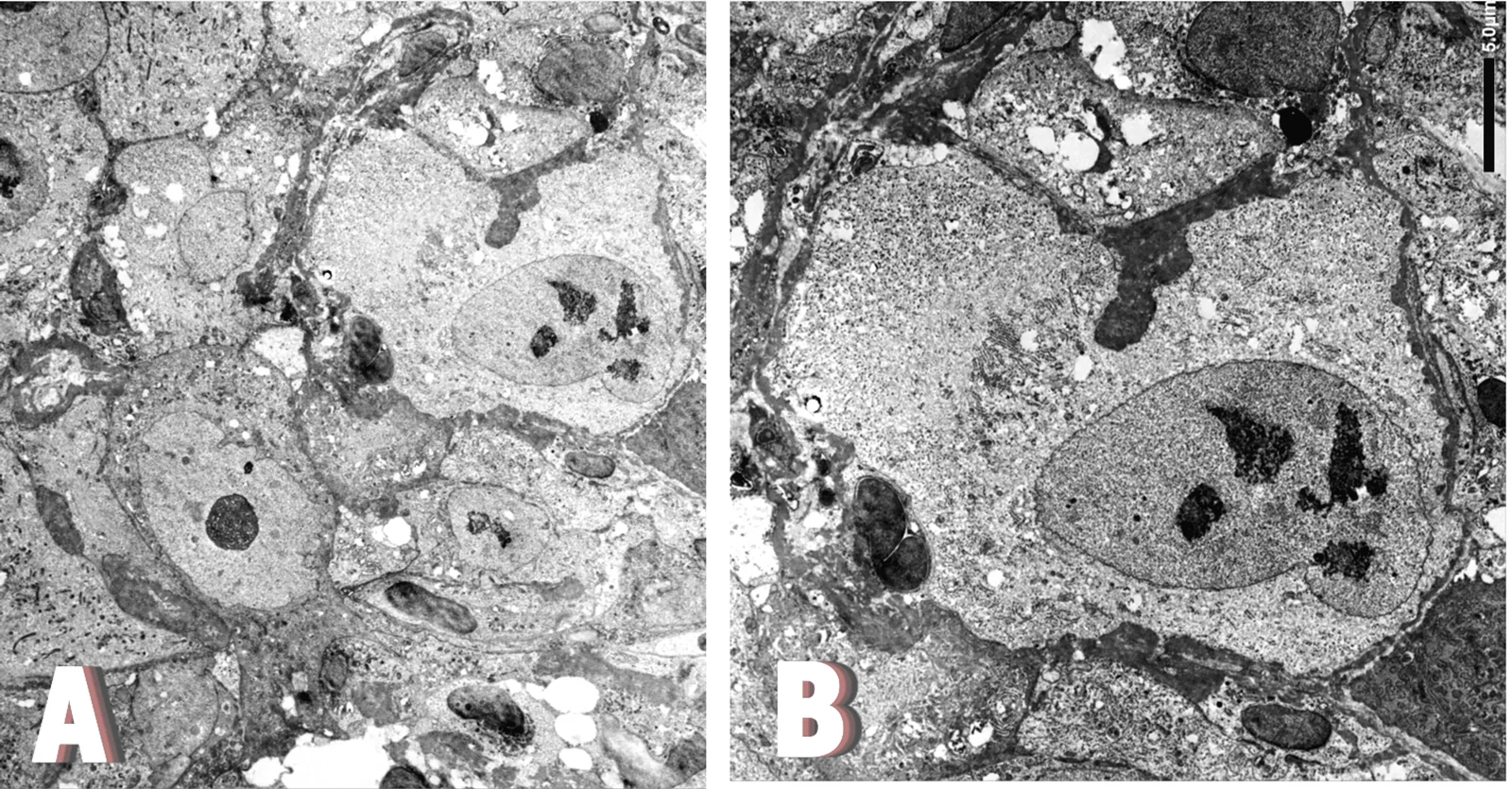
Ultrastructural features A, B: Large tumor cells of various shapes and sizes are in sheets, and euchromatic nuclei containing one or multiple prominent nucleoli. Intermediate filaments are scattered in the cytoplasm, but no microvilli are observed on the tumor cell surface. (A) Original magnification x 3,000. (B) Original magnification x 9,000.

Lesion tissues were fixed in paraffin blocks for evaluation using an electron microscope. The examination revealed findings similar to those of histological examination. Large tumor cells of various shapes and sizes were present in sheets, and the euchromatic nuclei contained one or more distinct nucleoli. Intracytoplasmic intermediate filaments were scattered in bundles; however, no microvilli were observed on the cell surface (Figure 4).

## Discussion

Mesotheliomas arising from the pleura, peritoneum, pericardium, and testicular capsule are classified as epithelioid, biphasic, and sarcomatoid mesotheliomas and can be difficult to diagnose because of their diverse histological features. Differential diagnoses include various primary tumors, metastatic tumors, and non-neoplastic lesions in the thoracic and abdominal cavities [1,4,6]. At present, immunostaining using calretinin, D2-40, WT-1, heart development protein with EGF-like domains 1 (HEG1), and epithelial membrane antigen (EMA) as positive markers as well as CEA, TTF1, MOC-31, and claudin 4 as negative markers, has contributed to improving the diagnosis of mesothelioma [7]. Mesothelioma has been diagnosed by the homozygous deletion of CDKN2A by FISH, so far. Recently, immunohistochemical methods using BAP1, MTAP, and Merlin/NF2 were developed as alternatives to FISH. When the presence of homozygous deletion of CDKN2A by FISH was used as the criterion, the specificity of BAP1, MTAP, and Merlin deficiencies for mesothelioma was 100% [1,4,8]. However, when differentiating mesothelioma from reactive mesothelial hyperplasia and benign mesothelial tumors, the sensitivity of BAP1, MTAP, and Merlin/NF2 deficiencies was approximately 50% individually, whereas the sensitivity of combined BAP1 deficiency with MTAP deficiency increased to approximately 80% and further increased to more than 95% when Merlin/NF2 deficiency was added. Thus, these combinations are extremely useful as an auxiliary diagnostic tool for mesothelioma [8]. In the present tumor, immunopositive reactions for calretinin, WT1, and D2-4, as well as loss of expression of BAP1, MTAP, and Merlin/NF2, and a high rate of homozygous deletion of CDKN2A by FISH were demonstrated. Therefore, these findings supported the diagnosis of mesothelioma. In contrast, in sarcomatoid mesotheliomas, the expression levels and frequencies of these markers decreased than those in epithelioid mesotheliomas. Thus, it may be difficult to distinguish between fibrous pleuritis and various sarcomas that present histologically similar features. In such cases, CDKN2A-FISH is extremely useful for differentiating these diseases [1,4,8].

The present case was a rare pleural deciduoid mesothelioma observed in an elderly man with a history of asbestos exposure. Electron microscopy revealed no microvilli on the surfaces of the tumor cells. After the first report of this tumor [5], it had been believed to arise from the peritoneum in young women with no history of asbestos exposure. However, as the number of cases increased, cases arising from the pleura, cases with a history of asbestos exposure, cases occurring in men, and cases occurring in elderly patients were reported, invalidating the previously believed characteristics [9-13]

Regarding the clinical findings of deciduoid mesotheliomas, Paliogiannis et al. reviewed 44 cases published in 16 articles on pleural cases. The patients' ages ranged from 13 to 78 years (mean 63 years), the male-to-female ratio was 1.87:1, and 58% had a history of asbestos exposure. The main symptoms were chest pain, dyspnea, cough, and weight loss [11]. Ordóñez performed a detailed study of 21 cases, including 17 pleural and 4 peritoneal cases, with a male-to-female ratio of 15:6 and an age ranging from 39 to 78 years (mean, 60 years). Histologically, the tumor cells were characterized as large epithelioid cells with clear borders, homogeneous eosinophilic cytoplasm, single or multiple atypical nuclei, and sheet-like or clustered growth patterns. The median survival time of the patients was 23 months. However, some cases showed reduced clustering of tumor cells, severe nuclear atypia, abnormal karyorrhexis, and abnormal mitosis. In such cases, the median survival time was only seven months, indicating a poor prognosis. In addition, electron-microscopic observations of nine cases showed long and dense microvilli on the surface of the tumor cells, and numerous intermediate filaments were present in bundles in the cytoplasm, confirming that the tumors were derived from mesothelial cells [10]. In previously reported cases, intermediate filaments have been detected in the cytoplasm, and some cases with long and dense microvilli [10] as well as short and sparse microvilli [5] have been reported. In contrast, intracytoplasmic intermediate filaments were observed in the present case, but microvilli were not present. The significance of such differences in microvillous structure among cases remains unknown; however, examining more cases with electron microscopy is expected to clarify this issue.

To the best of our knowledge, there are few case reports on the cytological findings of decidual mesothelioma, and no large-scale analyses are available. Reis-Filho et al. reported that, in a pericardial lesion of an elderly woman, the tumor cells were cytologically characterized by large polygonal shapes, homogeneous cytoplasm, pleomorphic, vesicular, atypical nuclei, and prominent nucleoli [14]. Huang et al. [15] described the cytological findings of peritoneal fluid from a male patient in his late teens with no history of asbestos exposure and an elderly woman with a history of asbestos exposure. The tumor cells of the former showed marked nuclear atypia and pleomorphism and were isolated and scattered, whereas those of the latter were multi-clustered with pseudo-acinar structures and intercellular fenestrae, accompanied by extracellular material, and showed marked nuclear pleomorphism. The authors stressed that the characteristics of mesothelial origin in both cytological findings were unclear and could be mistaken for other malignancies [15].

In the present case, pleural fluid cytology revealed that large epithelioid cells with atypical central nuclei containing single or multiple clear nucleoli appeared individually or in small clusters. Considering the clinical findings, including multiple pleural nodules on CT, pleural effusion, and an extremely high level of hyaluronic acid in pleural fluid, a cytological diagnosis of epithelioid mesothelioma was possible. However, as in previous reports, the histological type of the deciduoid mesothelioma could not be determined. In other words, a significant discrepancy existed between the cytological and histological features; in the cytological features, epithelioid tumor cells had single round or oval nuclei, while in the histological features, epithelioid cells of various sizes and irregular shapes had atypical, pleomorphic, or multinucleated nuclei. Although no complete explanation for the reason is available, one possibility is that the biopsy was taken from only one of the multiple lesions of the pleura, and other lesions may contain mesothelioma components that are not deciduoid mesothelioma. Therefore, the cytological findings in the present case may not reflect a decidual mesothelioma. However, further investigation is required to confirm this hypothesis.

To date, patients with mesotheliomas are usually treated with chemotherapy; however, their prognoses are poor. Recently, immune checkpoint inhibitors have emerged as a new treatment, and the administration is expected to improve the prognosis of mesothelioma as well [16-18]. However, because deciduoid mesothelioma is extremely rare, there are few reports of the administration of immune checkpoint inhibitors for this tumor, and their effectiveness remains unknown. In the present case, there was no deterioration of symptoms seven months after combination therapy with immune checkpoint inhibitors and chemotherapeutic agents. However, long-term follow-up is necessary to evaluate its effectiveness.

Recently, a human mesothelioma cell line, D-Meso-Sonobe, has been reported [19]. When transplanted into mice, this cell line forms tumors that exhibit characteristics similar to those of the rare decidual mesothelioma. Therefore, it is expected that the use of this cell line will not only allow for a detailed elucidation of the morphological and biological characteristics of this tumor, but also lead to the development of effective treatment [19].

## Conclusions

Here, we report a rare case of deciduoid mesothelioma in an elderly man with a history of asbestos exposure. Epithelioid mesothelioma was easily diagnosed based on the cytological findings. Histologically, the tumor was characterized by a sheet-like proliferation of large epithelioid tumor cells with severe nuclear atypia and pleomorphism. Immunohistochemical loss of expression of mesothelioma cell markers, including BAP1, MTAP and Merlin/NF2 as well as the homozygous loss of CDKN2A by FISH, were observed. These findings supported the diagnosis of deciduoid mesothelioma. Seven months after the initiation of combined therapy with immune checkpoint inhibitors and chemotherapeutic drugs, the disease was controlled; however, further long-term follow-up is required.
